# Modelling Species Selectivity in Rat and Human Cytochrome P450 2D Enzymes

**DOI:** 10.1371/journal.pone.0063335

**Published:** 2013-05-14

**Authors:** Grace H. C. Edmund, David F. V. Lewis, Brendan J. Howlin

**Affiliations:** Department of Chemistry, Faculty of Engineering and Physical Sciences, University of Surrey, Guildford, Surrey, United Kingdom; Concordia University Wisconsin, United States of America

## Abstract

Updated models of the Rat Cytochrome P450 2D enzymes are produced based on the recent x-ray structures of the Human P450 2D6 enzyme both with and without a ligand bound. The differences in species selectivity between the epimers quinine and quinidine are rationalised using these models and the results are discussed with regard to previous studies. A close approach to the heme is not observed in this study. The x-ray structure of the enzyme with a ligand bound is shown to be a better model for explaining the observed experimental binding of quinine and quinidine. Hence models with larger closed binding sites are recommended for comparative docking studies. This is consistent with molecular recognition in Cytochrome P450 enzymes being the result of a number of non-specific interactions in a large binding site.

## Introduction

Human cytochrome P450 2D6 (CYP2D6), part of the cytochrome P450 (CYP450) superfamily of heme containing enzymes, plays an important role in the phase I mono-oxygenase metabolism of xenobiotic substrates being responsible for the metabolism of ≈20% of therapeutic drugs in current clinical use [1].
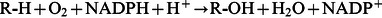
(1)


The importance of CYP450s to drug metabolism has long made them a target for investigation e.g. oxidation by CYP450s can activate prodrugs to their therapeutically active form, while their wide substrate specificity can result in drug-drug interactions, often detrimental to patient health. This is particularly obvious in the case of the CYP2D family, which due to its highly polymorphic nature can have a great deal of variability in terms of its metabolism. Better understanding of the molecular determinants of reactivity and specificity is therefore required.

Traditionally, animal models are used to predict a drug’s ADME-Tox properties, with the rat being one of the most common animal models used. However, there are questions regarding the transferability of these models to Human. Rats, after all, have 6 CYP2D enzymes; CYP2D1-2D5 and CYP2D18, compared to only one in humans, CYP2D6. The rat and human CYP2D isoforms show a reasonably high sequence identity overall (≈56%) but this is significantly lower in the active site region (≈34%).

**Figure 1:**
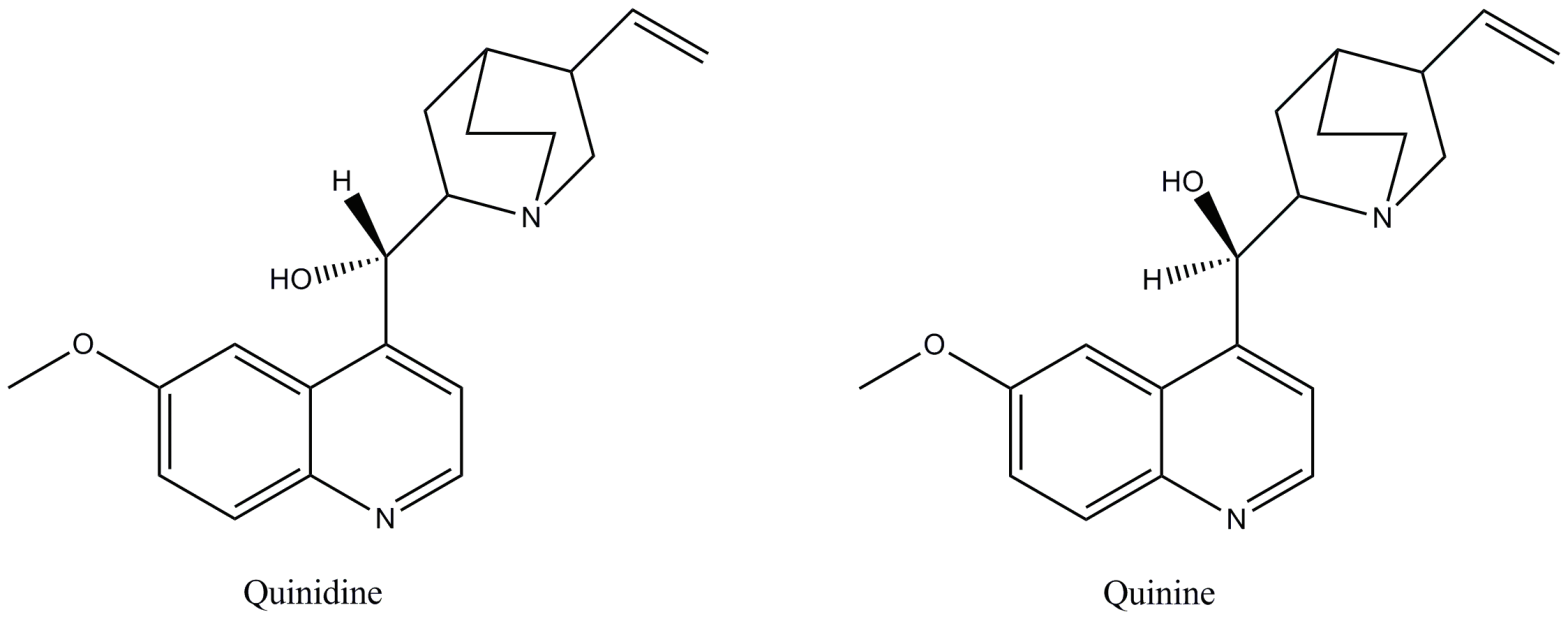
Structures of the epimers quinine and quinidine.

A key example of this is the observed species difference is the effect of chirality on CYP2D6. The chiral enantiomers of quinidine and quinine show a species selective response when metabolised by either human or rat (Figure 1). Quinidine is observed to be a strong inhibitor of human CYP2D6, while quinine, although having no effect on human CYP2D6 metabolism is a strong inhibitor of rat CYP2D enzymes, especially rat CYP2D2 (Table 1 and Table 2).

**Table 1:** Experimental IC_50_ values for quinidine and quinine in rat and human CYP2D taken from Venhorst *et al*. [1].

IC_50_/µM					
Ligand	CYP2D1	CYP2D2	CYP2D3	CYP2D4	CYP2D6
Quinidine	19.9±1.9	2.8±0.7	26.9±4.4	47.2±13.4	0.0033±0.001
Quinine	46.5±7.6	0.0094±0.009	12.0±0.2	1.7±0.4	0.61±0.05

**Table 2:** Experimental and Calculated K_i_ values for quinidine and quinine in both crystal structures of human CYP2D6.

Ligand	ExperimentalK_i_/µM	CalculatedK_i_/µM
Quinidine Crystal Structure	0.03	2.13×10^−7^ (2F9Q)
		5.07×10^−9^ (3QM4)
Quinidine Molecular Structure	0.03	2.65×10^−8^ (2F9Q)
		2.01×10^−8^ (3QM4)
Quinine	5.9	1.41×10^−6^ (2F9Q)
		2.77×10^−7^ (3QM4)

Previous modelling work on Cytochrome P450 has been carried out by Lewis, see for example [2] and references therein. Recently Zhang has published a machine learning model of species selectivity [3] which has a greater than 80% accuracy in predicting selectivity. However, models of this kind do not provide information on the three dimensional structure of the active sites of the proteins nor the interactions made by the ligands. Similarly, there have been many studies that have used quantitative structure activity relationships [2] to probe ligand binding but again these studies lack 3 dimensional information. Mulholland et al. [4] have used the combined techniques of QM/MM to study the interactions with the iron in CYP, which is the currently highest level of modelling that can be achieved in proteins and have been useful in describing the mechanistic details of the catalytic cycle. Venhorst et al. [1] have generated homology models based on the x-ray structure of rabbit CYP2C5 and used this to identify 22 active site residues that are of importance in ligand binding. In this work we have based our models on the now available x-ray structures of the Human CYP2D6 which is a better starting point for the comparison. Computational techniques, such as homology modelling and molecular docking can be used to effectively investigate any sequence variation between species. This work describes a variety of techniques used to create homology models of rat CYP2D and to investigate the cause of species selectivity in Rat and Human enzymes.

Previous work on molecular recognition in antibody:antigen interactions concluded that the ability of antibodies to recognise a wide variety of ligands was not due to specific interactions with certain key selected amino acids but rather was due to a range of non-specific interactions in the overall active site [5]. CYP450’s also show this inducible nature where they can also respond to a wide variety of ligands and one of our aims in this paper was to see if, with ligands that differ only in the arrangement about one chiral centre, quinidine and quinine would show specific interactions or would also show non-specific binding.

## Methodology

### Homology Models of Rat Cytochrome P450 2D Enzymes

**Figure 2:**
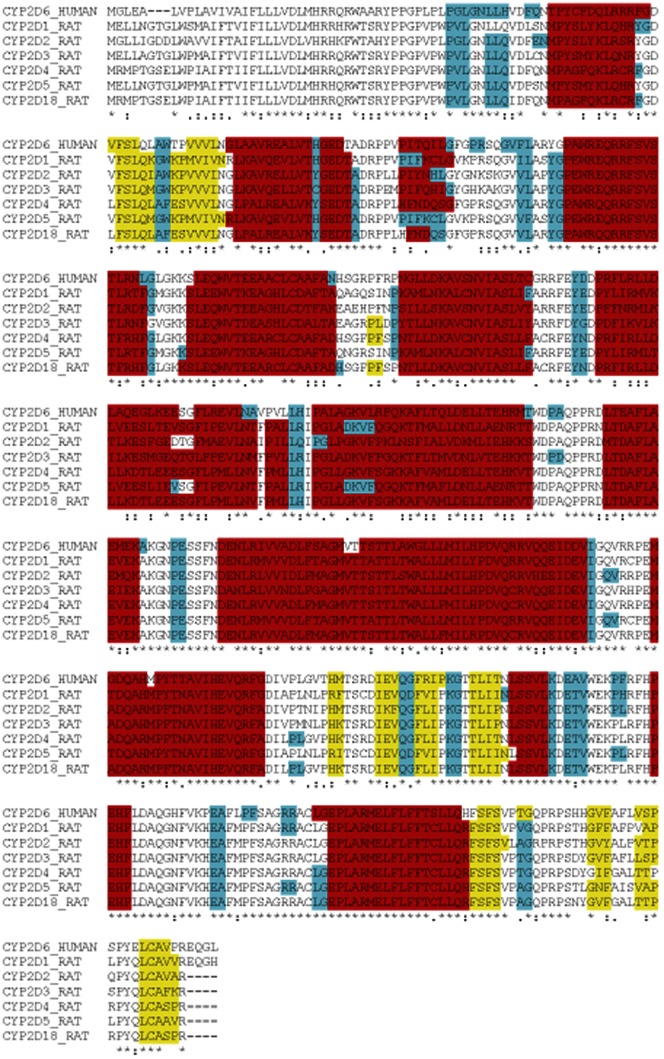
Sequence Alignment of Human CYP2D6 and all Rat CYP2D enzymes. α-helices are shown in red, β-sheets in yellow and β-turns in blue. The sequence alignment was generated using both ClustalX and the MOE sequence alignment program.

Full length sequences of human and rat CYPs were taken from the UniProt database [6] with a combination of PSIPred [7], JPred [8] and Porter [9] being used to predict the secondary structure of the rat CYPs. A series of sequence alignments were generated using ClustalX [10], [11] based on all known human and rat CYP2D sequences (Figure 2). The predicted secondary structure from PSIpred, JPred and Porter disagreed at one point with that predicted from ClustalX. Hence a manual modification of the sequence alignment was performed to give the best consensus match with the highest sequence identity (≈57% between all rat CYP2D enzymes and ≈56% between rat and human CYP2D enzymes). The Molecular Operating Environment (MOE) [12] was then used to generate 25 high-precision, three-dimensional homology models for rat cytochrome P450 2D1–5 and 2D18 enzymes. The models were based on the available human CYP2D6 crystal structures both with a ligand bound in the active site (PDB entry code 3QM4 [13]) and without (PDB entry code 2F9Q [14]). Models were generated under both the Amber99 and Charmm27 force fields and all relevant crystallographic information was retained. The models formed were analysed both visually and statistically within MOE [12].

### Molecular Dynamics Studies on Homology Models

MOE [12] was used to perform a series of molecular dynamics (MD) simulations *in vacuo* to judge the stability and quality of the homology models generated. The simulations were performed using the NPT ensemble, which holds the number of atoms, the pressure and the temperature constant throughout the simulation while allowing volume to vary. This ensemble was chosen as the simulations were performed at body temperature and pressure which is not normally subject to change in biological systems. Simulations were run at both 0 K and 0 Pa for 2 ps and 310 K and 101 Pa for 2 ps.

### CYP2D Active Site Studies

Site Finder [15] available within MOE [12] was used to generate a series of possible ligand binding sites within the two human CYP2D6 crystal structures, by identifying clusters of relevant ‘alpha spheres’ considered to be in solvent accessible regions of hydrophobicity and hydrophilicity [16], [17]. A surface analysis of the active sites of both human CYP2D6 crystal structures was performed using the surface mapping tool available within MOE [18], [19], [20] with the active site definitions being taken from literature [1] and the Site Finder [15] analysis.

### Inhibitor Docking

Quinidine and quinine, which are known inhibitors of human Cytochrome P450 2D6 were docked into both the human CYP2D6 crystal structures [13], [14] and the rat CYP2D homology models using the available MOE docking program. In previous work from our group [21] results more consistent with experiment have been achieved by docking the x-ray crystal structure of the ligand rather than a model built up on an atom by atom basis. Therefore we have docked both the x-ray crystal structure of quinidine [22] and the molecular model of quinidine and the model of quinine as there is no x-ray structure of quinine available.

Docking was performed using three methods; an alpha triangle rigid-protein-flexible-ligand dock with a force field based refinement step and London ΔG scoring functions, an alpha triangle rigid-protein-rigid-ligand dock with a Gridmin based refinement step and London ΔG scoring function and a manual placement and force field based minimisation step. As the force field based methods caused some anomalies to occur with the aromatic rings of the ligands, the Gridmin based docking method was used as this greatly reduced the number of observed anomalies in this case. The manual placement method was used to see if interaction with the Fe atom was possible with these ligands. In practice as these ligands are inhibitors and not substrates the Gridmin results were taken as less subject to bias on our behalf and none of these dockings showed a close approach to the Fe atom. The difference in docking behaviour between substrates and inhibitors will be the subject of the further publication.

For each of the docks, the following information was recorded for each contact made with the protein (more than one in some cases):

- Residue types, number and subunit
- Bonding type (backbone acceptor/donor, H-bond acceptor/donor (side chain));
- Distance between donor and acceptor as calculated by MOE (Å);
- Contact point on the ligand according to the numbering scheme.

### Ligand Optimisation

In order to correct for the anomalies in aromatic structure observed during docking, a ligand optimisation script provided by MOE [12] was employed. This script duplicated the docked ligand and corrected the confirmation before refining the duplicate on to the original confirmation. This step was only required for the docks of quinidine and quinine in the 2F9Q based models of CYP2D2 and for quinidine in CYP2D5. No such anomalies were observed when docking to the 3QM4 based structures.

### Protein-Ligand Binding Analysis

A combination of the 3D ligand interaction prediction and 2D ligand interaction tool from MOE were used to predict and visualise protein-ligand interactions likely to occur for each of the bioactive compounds docked.

### Induced Docking Model

Owing to the inducible nature of CYP2D enzymes a further energy minimisation step was introduced into the docking analysis in order to more accurately model enzyme response. After docking the protein was minimised under the CHARMM27 force field [23] which is parameterised for protein simulations around the fixed heme and docked ligand providing a representation of the induced docking model.

### Calculation of K_i_ from ΔG

K_i_ values were calculated for each of the docked compounds for comparison with experimental values (Table 1 and Table 2). K_i_ values were calculated from ΔG using;
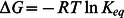
(2)

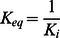
(3)

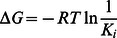
(4)

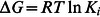
(5)

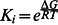
(6)


## Results and Discussion

**Figure 3:**
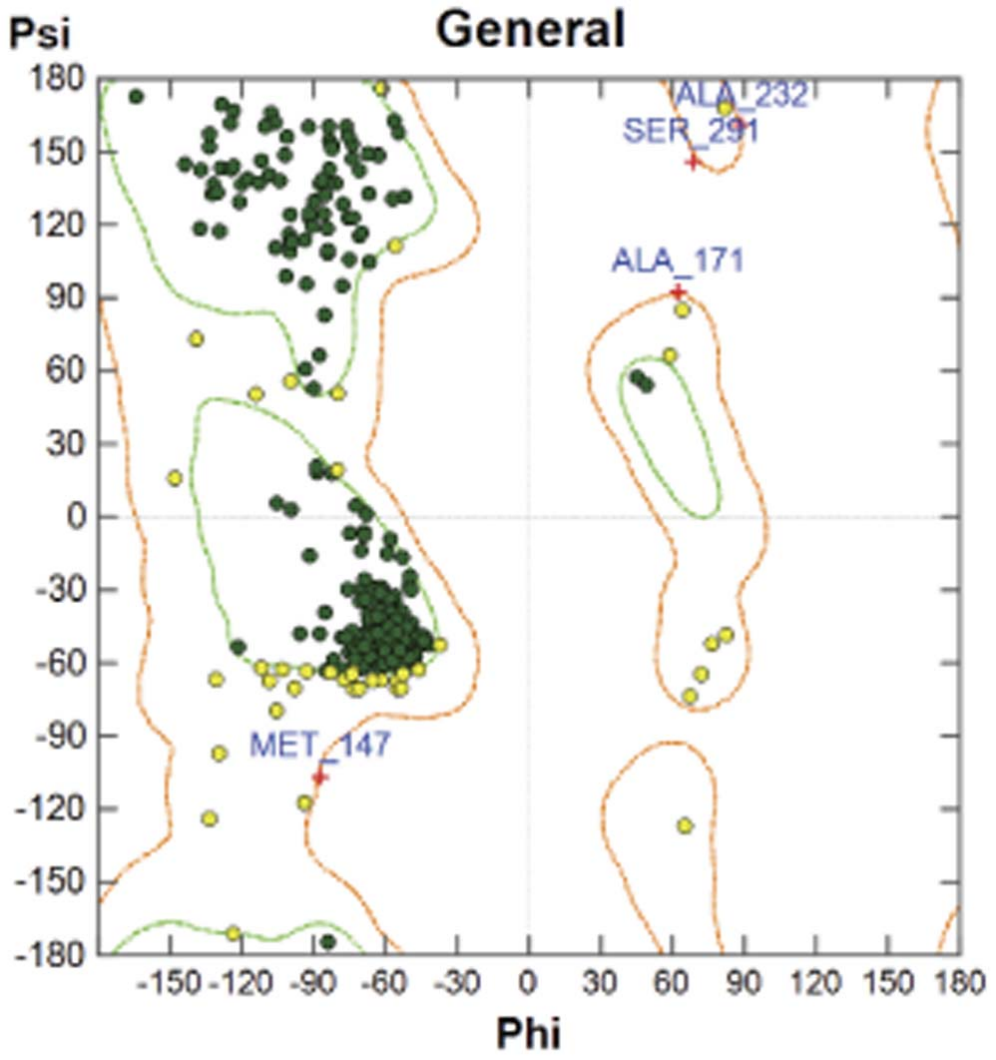
Ramachandran Plot for a model of rat CYP2D1 based on 2F9Q.

**Figure 4:**
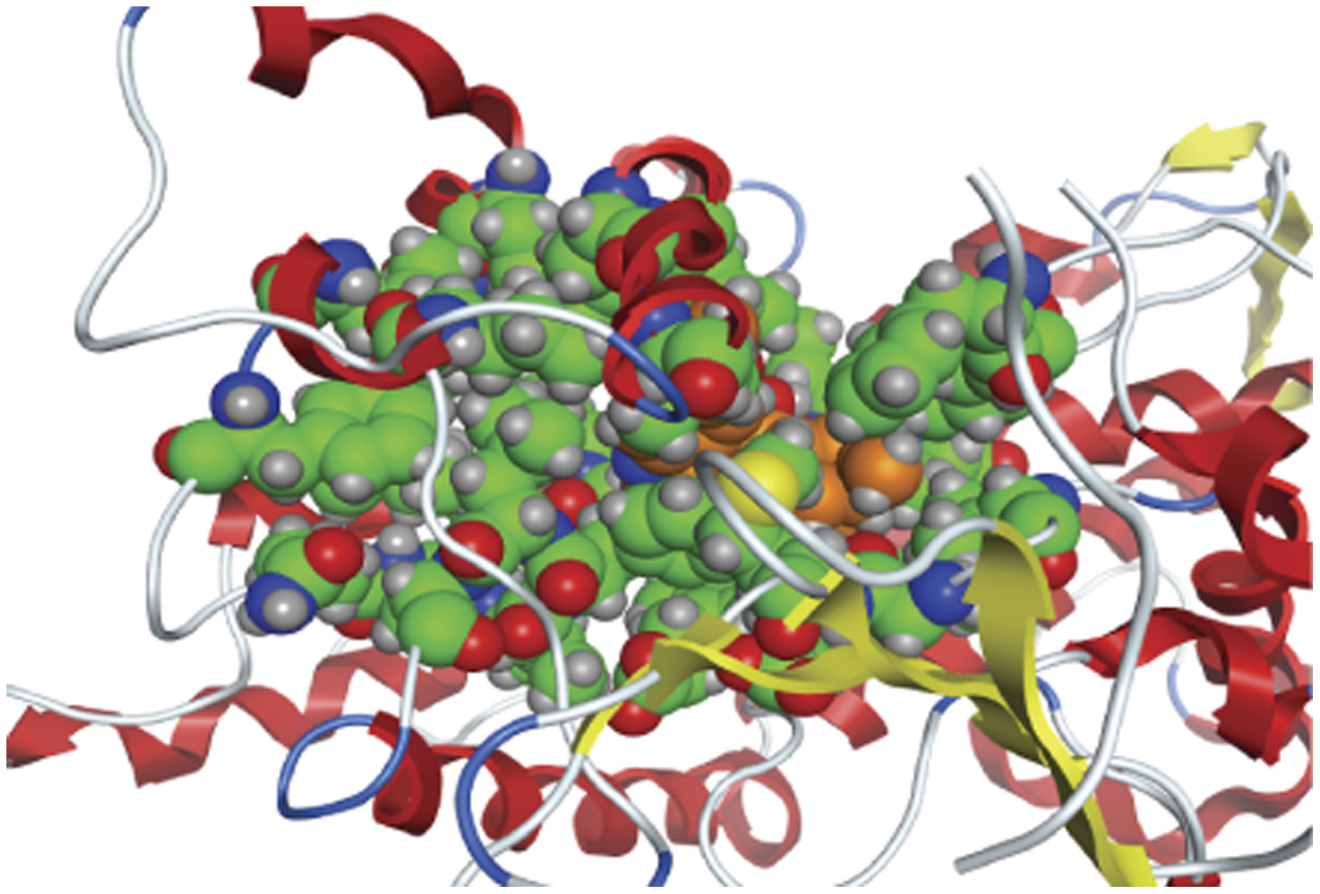
Quinidine inducibly docked into a model of human CYP2D6 based on the 2F9Q crystal structure. Quinidine is shown in orange inside the active site which is shown in green.

**Figure 5:**
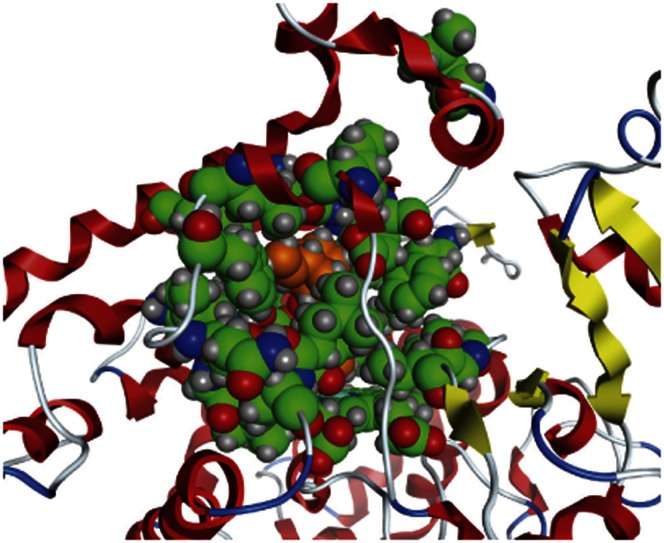
Quinidine inducibly docked into a model of human CYP2D6 based on the 3QM4 crystal structure. Quinidine is shown in orange inside the active site which is shown in green.

**Figure 6:**
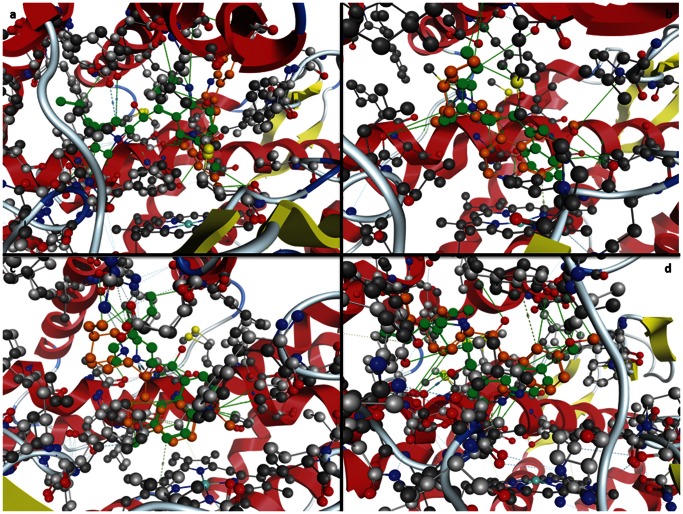
Interaction diagram showing an overlay of quinidine (green) and quinine (orange) in a) Human 2D6 (2F9Q), b) Rat 2D2 (2F9Q), c) Human 2D6 (3QM4), d) Rat 2D2 (3QM4). The lines shown in green are electrostatic interactions (pi bonding or H bonding).

The homology models were analysed by Ramachandran plots (Figure 3). This clearly shows that 99% of the residues are in allowed conformations and there are no outliers, providing confidence in the models used. The x-ray structure of prinomastat bound to Cytochrome P450 2D6 [14] was used to validate the docking procedure. The docking results showed that MOE was able to reproduce the x-ray structure with an rmsd of 0.5592 Å and that prinomastat was in the vicinity of Ser304 and Glu219. There is no interaction in this x-ray structure with Asp301, which has been proposed as a vital residue [24]. One of the major concerns in cytochrome P450 modelling is the inducibility of the active site [25] i.e. if one compares the x-ray structures of the native Human 2D6 with the structure with prinomastat bound there is a significant difference (increase in volume of 130 Å) in the active sites. The active site of the ligand bound structure is larger but more closed indicating that the protein has conformed to the shape of the ligand. Therefore we have carried out docking to both the homology models of the native enzyme and the ligand bound models (Figures 4 and 5). Table 3 shows that there are more interactions with amino acid residues in all models for those based on 2F9Q rather than 3QM4 indicating that the active site is smaller as discussed above. Table 3 shows the close approaches (within 3.8 Å) of amino acid residues in the rat and human models (based on 2F9Q and 3QM4) with both quinine and quinidine. Figure 6 shows a visual representation of the interactions summarised in Table 3, which makes the data presented easier to interpret. In the models based on the 2F9Q structure interactions with residue 304 occur for all rat and human models for both ligands. Residue 213, 216, 244 and 301 are shown to interact with both human and rat models in quinidine but not in quinine, whereas quinine interacts with 308, 374 and 483 in both human and rat but not in quinidine. Interactions that occur in both human and rat models indicate similar modes of binding in both models. There are ten interactions with the Human 2D6 model in quinidine but only seven in quinine. Of these only four are common, i.e. those with 217, 220, 304 and 373. In the models based on 3QM4, residues 216, 244, 301, 304 and 309 show interactions with both ligands in both human and rat structures with almost all rat and human models showing interactions with 301 for both ligands. There are ten observed interactions with quinidine and nine with quinine in the human structure. Of these interactions seven are common including residues 117, 216, 244, 301, 304, 309 and 484. Previous work on the docking of quinine and quinidine using the models based on the rabbit x-ray structure [1] indicated the quinidine interacted with Asp301, Ser304 and Phe120 in the human model and couldn’t approach Asp301 as closely in the Rat 2D2 model but formed a hydrogen bond to the main chain of Met304 (replaces Ser304 in 2D2). In this work, interactions between 2D2 and Asp301 are found with models based on 2F9Q showing a movement of the Met304 side chain during the energy minimisation step to allow for interaction between quinidine and Asp301. For those models based on 3QM4 the Met304 side chain is already in a position which allows for interaction with Asp301. In the previous work on the quinine docking [1], interactions were found to the carbonyl backbone of 301 in the human model and to Asp216, Thr217 and Met304 in the rat 2D2 model. Interactions were found to backbone and side chain of Asp301 in the 3QM4 and 2F9Q human structures respectively, but neither Asp216 nor Met304 interactions were observed for rat 2D2.Experimental binding data for Human 2D6 (Table 1 and Table 2) shows quinine binds approximately 1000 times less well than quinidine. In both the 2F9Q and 3QM4 based models of human 2D6, more interactions were observed for quinidine than quinine. However, the experimental binding situation is reversed in the Rat 2D2 where quinine binds approximately 1000 times tighter than quinidine. Here the quinine should make more contacts with the protein. This is seen in the 3QM4 model where ten contacts are observed for quinine while quinidine has only nine. In fact these results follow the experimental data in most cases with more interactions being observed for the more potent epimer for all but the 2F9Q based model of 2D2 and the 3QM4 based model of 2D1. If the lowest energy docking results are considered (Table 4), in the models based on the 2F9Q structure for the 2D2 case the quinine has a lower free energy of binding than the quinidine model structure, −11.68 to −9.8 Kcal/mol. The results are reversed when the quinidine x-ray structure is compared to the quinine model structure, indicating that there is no advantage in using the x-ray structure in this case. In the 2D6 model based on 2F9Q, it is the quinidine model that has the lowest binding free energy. This is consistent with the experimental results. For the models based on the 3QM4 structure the quinidine (X-ray or model structure) has a lower free energy of binding than the quinine in all cases. This is only consistent with experiment for the 2D6 case, so these results cannot explain the difference observed in the 2D2 case. Therefore, in the case of simulated binding free energies, the models based on the open active site are more consistent with experiment. In the previous work [1], the differences in binding efficiency between the epimers were ascribed to the difference in close approach to the iron atom in the heme. However, it was stated that the ligands were located close to the heme in this work but that this was not a requirement for all competitive inhibitors, like these ligands, and multiple binding modes are possible. These large differences in experimental binding efficiency cannot be prescribed wholly to small differences in interaction energies, so as in the antibody:antigen case, it is the sum total of the weak interactions overall that account for the inducible nature of CYP450 binding and this is true in the qunine:quinidine case also. The large non-specific binding site of CYP450 is ideally suited to binding a wide variety of ligands.

**Table 3:** Amino acid residue interactions with Quinidine and Quinine in models based on both crystal structures.

*Ligand*	2F9Q		3QM4	
*Residue Number*	Quinidine	Quinine	Quinidine	Quinine
**54**	2D18			
**107**			2D2, 2D3 (π)	2D2 (π)
**110**				2D2
**112**			2D2, 2D3	2D2
**117**			2D6, 2D6 (π)	2D6 (π)
**120**	2D6		2D1 (π), 2D2 (π)	2D1, 2D4, 2D4 (π), 2D18, 2D18 (π)
**121**	2D3, 2D4	2D18	2D2	2D2, 2D3, 2D18
**208**			2D6	
**209**			2D6	
**212**			2D3	
**213**	2D2, 2D3 (π), 2D6, 2D18	2D1, 2D3, 2D4, 2D5, 2D5 (π)	2D3	
**216**	2D2, 2D3, 2D6	2D1, 2D2, 2D3	2D1, 2D2, 2D4, 2D5, 2D6, 2D18	2D2, 2D4, 2D5, 2D6, 2D18
**217**	2D2, 2D3, 2D4, 2D6	2D3, 2D5, 2D6, 2D18		
**220**	2D2, 2D3, 2D4, 2D6, 2D18	2D2, 2D6, 2D18, 2D18 (π)		
**244**	2D1, 2D5, 2D6	2D3	2D1, 2D3, 2D6	2D1, 2D2, 2D5, 2D6, 2D18
**248**		2D2		
**297**			2D2, 2D4	2D3, 2D4
**300**	2D6	2D3	2D1, 2D4, 2D18	2D1, 2D2, 2D3, 2D4, 2D5, 2D6, 2D18
**301**	2D1, 2D2, 2D6	2D1, 2D2, 2D3	2D1, 2D2, 2D3, 2D4, 2D5, 2D6, 2D18	2D1, 2D3, 2D4, 2D5, 2D6, 2D18
**304**	2D1, 2D2, 2D3, 2D4, 2D5,2D6, 2D18	2D1, 2D2, 2D3, 2D4, 2D5, 2D6, 2D18	2D2, 2D4, 2D6, 2D18	2D1, 2D2, 2D4, 2D6, 2D18
**305**	2D3, 2D4, 2D18	2D2, 2D3, 2D18	2D2, 2D4	2D1, 2D2, 2D3, 2D4, 2D18
**308**	2D2, 2D18	2D3, 2D5, 2D6, 2D18	2D18	2D6
**309**	2D1, 2D2, 2D3	2D1, 2D3, 2D4	2D1, 2D5, 2D6	2D3, 2D6
**369**		2D1		
**370**	2D2, 2D18	2D2, 2D4	2D3, 2D5	
**372**	2D18	2D4, 2D18		
**373**	2D1, 2D2, 2D4, 2D5,2D6, 2D18	2D1, 2D4, 2D6, 2D18	2D5, 2D18	2D1, 2D3, 2D18
**374**	2D1, 2D1 (π), 2D2, 2D3, 2D18	2D2, 2D3, 2D6, 2D18 (π)		2D1, 2D5
**375**	2D4			
**482**	2D18			
**483**	2D3, 2D4, 2D5, 2D18	2D1, 2D4, 2D5, 2D6, 2D18	2D5	2D1, 2D2 (π), 2D18
**484**	2D1, 2D5, 2D18 (π)	2D3, 2D4, 2D5	2D6, 2D18	2D6

**Table 4:** ΔG_binding_ energies calculated from docking simulations to CYP2D2 and CYP2D6 models.

Ligand	2F9Q					
	CYP2D2			CYP2D6		
	E (kcal/mol)	Ki	LogKi	E (kcal/mol)	Ki	LogKi
Quinidine Crystal Structure	−13.8513	6.93826E-11	−10.1587492	−13.3215	1.69752E-10	−9.770186008
Quinidine Molecular Structure	−9.8005	6.48885E-08	−7.187832299	−10.4836	2.04726E-08	−7.688827987
Quinine	−11.6875	2.68049E-09	−8.571786133	−10.9867	8.75369E-09	−8.057809001
	**3QM4**					
	**CYP2D2**			**CYP2D6**		
	**E (kcal/mol)**	**Ki**	**LogKi**	**E (kcal/mol)**	**Ki**	**LogKi**
Quinidine Crystal Structure	−11.3477	4.75804E-09	−8.322571765	−12.047	1.46067E-09	−8.835448774
Quinidine Molecular Structure	−11.3699	4.58296E-09	−8.338853574	−11.2423	5.685E-09	−8.245269839
Quinine	−10.4934	2.01365E-08	−7.696015453	−11.0223	8.24293E-09	−8.08391857

In ligands with polar groups prominent there is an interaction observed with the iron of the Heme in the protein. This is consistent with the x-ray results for prinomastat which shows a Fe-N interaction with the pyridinyl ring. However, we do not observe a close approach to the iron in our docking studies. This may suggest that as both quinine and quinidine are inhibitors (at least *in vitro* [26]), their roles are to occupy space in the binding site thereby denying other ligands access to the active site.

### Conclusions

The basis of species selectivity in human and rat cytochromes is a complex problem, which demonstrates clearly how non-specific binding can be used in nature to ‘engineer’ proteins that are able to bind to a wide variety of substrates. An induced fit minimisation step is shown to be useful in reproducing experimental docking results. Hence models based on the larger closed active site in the 3QM4 structure are better models of the observed experimental data for quinidine and quinine than those based on the more open 2F9Q and that there are differences in both the number of interactions made by different ligands as well as their close approaches and it is important to take this into account when seeking to understand their binding. In all of the rat models studied this is true apart from 2D1 where the 2F9Q model is better at reproducing the experimental binding data.
